# Clinical Management of Long-Term Survivors after Classical Hodgkin Lymphoma and Diffuse Large B-Cell Lymphoma

**DOI:** 10.3390/cancers14235960

**Published:** 2022-12-02

**Authors:** Alessia Bari, Chiara Gerardi, Eleonora Allocati, Agata Puzzovivo, Vitaliana De Sanctis, Alessandra Tucci, Monica Balzarotti, Silvia Franceschetti, Francesco Merli, Attilio Guarini, Guido Gini, Carla Minoia

**Affiliations:** 1 Dipartimento di Scienze Mediche e Chirurgiche, Materno-Infantili e dell’Adulto, Università di Modena e Reggio Emilia, 41124 Modena, Italy; 2 Istituto di Ricerche Farmacologiche “Mario Negri” IRCCS, 20156 Milan, Italy; 3 Cardioncology Unit, IRCCS Istituto Tumori “Giovanni Paolo II”, 70124 Bari, Italy; 4 Department of Medicine, Surgery and Translational Medicine, “Sapienza” University of Rome, Radiotherapy Oncology, St. Andrea Hospital, 00189 Rome, Italy; 5 Hematology Department, ASST-Spedali Civili, 25123 Brescia, Italy; 6 Hematology Unit, IRCCS Humanitas Clinical and Reserach Hospital, Rozzano, 20089 Milan, Italy; 7 Haematology Unit, Ospedale Civile di Legnano, ASST Ovest Milanese, 20025 Legnano, Italy; 8 Hematology Unit, Azienda Unità Sanitaria Locale-IRCCS di Reggio Emilia, 42123 Reggio Emilia, Italy; 9 Hematology Unit, IRCCS Istituto Tumori “Giovanni Paolo II”, 70124 Bari, Italy; 10 Clinic of Hematology, AOU Ospedali Riuniti, 60126 Ancona, Italy

Compared to other patients suffering from hematological malignancies, classical Hodgkin lymphoma (cHL) and diffuse large B-cell lymphoma (DLBCL) patients have a long life expectancy when in complete remission at the end of first, or sometimes second, line treatments. Thus, after 5 years without relapse, they could be considered cured, and follow-up programs must be implemented to support these patients as long-term survivors. The aims of these programs are to prevent, rapidly diagnose and monitor late sequelae of lymphoma treatment, as well as to improve quality of life (QoL). 

An evidence-based approach to the long-term follow-up of cHL and DLBCL survivors is still an unmet clinical need [1], with specific guidelines and research based on treatments received and peculiar disease history lacking, differently from solid tumor.

In 2019, a multidisciplinary research team from the Fondazione Italiana Linfomi (FIL) proposed a series of systematic reviews to better understand the best applicable model for clinical management of long-term survivors of cHL or DLBCL. The aim was to highlight the gaps in long-term monitoring and follow-up and provide suggestions for further research in the field.

The working group included eight onco-hematologists, two cardiologists, two radiotherapists, a gynecologist, an endocrinologist, a psychologist and a nutritional biologist supported by a method team, comprising three experts in clinical research methodology and systematic reviews.

The project focused on cardiological, endocrine–metabolic, neurological/cognitive and psychological disorders, secondary cancers, fertility preservation and lifestyle, which are relevant matters for cancer survivors. Following a common methodology [2], six working groups conducted and published a series of systematic reviews focused on three main aspects: incidence of long-term toxicity, comparison of old or standard therapies with more recent ones and evidence of specific follow-up approaches.

The result of this collaborative effort manifested as six different manuscripts published as systematic reviews of evidence-based clinical management. The main areas covered included late toxicity, conditions and follow-ups in long-term survivors of cHL or DLBCL, in addition to highlighting the need to develop specific research programs. This collection could support decision makers in preparing sustainable evidence-based guidelines tailored for long-term survivors, such as the Survivorship Care Program (SCP).

With regards to long-term cardiovascular toxicity, the available evidence from dosimetric studies highlights that new radiotherapy (RT) techniques reduce the risk for late cardiovascular disease (CVD). Many indications for early detection and monitoring of secondary CVD derive from retrospective or uncontrolled studies instead. Clinical research and data supporting definitive recommendations for various tests and their frequency during follow-up of long-term lymphoma survivors still need to be generated by well-designed clinical trials. Further studies are also needed for the detection of cardiotoxicity predictive scores in lymphoma survivors and the design of personalized surveillance, which could be translated into improved patient outcomes. Tailored screening and prevention programs may be warranted to offset the future burden of disease [3]. 

Due to the restrictive inclusion criteria based on the methodology followed, we included only a few papers on the endocrine and metabolic sequelae. Nevertheless, we were able to extract strong data on the actual incidence of endocrine and metabolic sequelae, which were considerable even if patients were treated with modern therapeutic approaches, and the possibility of dedicated follow-up programs. For example, thyroid disfunction represents a late effect of neck RT in patients treated with intensity-modulated radiation therapy and a smaller radiation field. Other clinical issues reported are bone dysfunction and sarcopenia, both of which were detected in this series of patients that represent pathological conditions with a negative impact on QoL. Very few data are available on follow-up programs with a focus on the detection of endocrine and metabolic sequelae in lymphoma survivors. The lack of information on follow-up programs should prompt the development of follow-up schedules to record early endocrine and metabolic sequelae based on a multidisciplinary approach [4].

Moreover, the related neurological toxicity and cognitive long-term side effects and sequelae are difficult to detect and approach. Concerning neurotoxicity, long-term sequelae do not seem to be a particularly disabling problem, but the effects of specific drugs still need to be continuously monitored in the future so as not to risk underestimating the problem. Through a systematic review, the authors were able to confirm an increased incidence of psychological impairment in long-term cHL survivors, emphasizing its detrimental effect on QoL [5]. In particular, younger cHL survivor populations suffer more from the effects on regular development, relationships and social life. However, the majority of studies addressing cognitive functions, mood disturbances and fatigue were based on a subjective evaluation through self-assessment tools. This could be a limitation. The relevance of this issue will require further assessment through the involvement of a neuropsychologist for a more objective evaluation. Recognizing the psychological impact of chemotherapy on long-term survivors may raise physicians’ awareness of these psychological aspects during the active disease period and treatment and thus lead to greater prevention efforts. 

The issue of secondary cancers (SCs) in highly curable lymphoma, such as cHL and DLBCL, is a historical challenge. This issue has gained further attention with a new treatment approach which guarantees a higher probability of curing the disease and, as a consequence, a higher number of patients at risk of SC. In a systematic review, the incidence of SC was found to be higher in cHL and DLBCL patients treated with standard first-line treatment than in the general population, and increased over time [6]. As expected, the use of intensified regimens and autologous stem cell transplants increased the risk of SC in both patient populations, and was augmented by RT exposure. 

A special focus was dedicated to secondary breast cancer in cHL survivors, which decreased over time in patients treated with reduced radiation volumes and doses. The correlation between the use of dose/volume reduction with current technological instruments and the reduced incidence of SC can only be partially explained by the dosimetric studies we found. Thus, a consensus has yet to be reached. 

From a more general point of view, in the context of screening tools and programs for the early detection of primary cancers applied to the general population, lymphoma survivors represent a population at higher risk that could benefit from a tailored screening program for SC according to their individual risk profile (family history, age, sex, type of anticancer treatment). Planning such a challenging screening program should engage not only (and not mainly) the treating onco-hematologist but also the general practitioner and other specialists involved in SC identification in a large national health system multidisciplinary program.

One of our systematic reviews addressed the topic of fertility. As infertility is a relevant complication of chemo-radio treatment, this issue must be discussed at the time of diagnosis, particularly with patients of childbearing age [7]. Regimens containing alkylating agents are known to have deleterious effects on both spermatogenesis and ovarian reserve. Gonadotoxicity of non-alkylating agents is less frequent, while the toxicity of recently introduced new drug is still unknown. In addition, RT of the pelvis, with/without chemotherapy (CT), may lead to severe injury to the ovarian reserve or testicular germ cells. Many efforts have been made to improve fertility preservation procedures. Obviously, the gonadotoxicity risk is strongly related to the type and stage of lymphoma and, consequently, to the type and the dosage of anticancer treatment as well as the age of female patients. Sperm cryopreservation before lymphoma treatment is the most efficient and widely available method used for fertility preservation in males, with no upper age limit. Approximately 80% of lymphoma survivors experience impairment of spermatogenesis, and 40% of patients who wish to have children are not able to conceive spontaneously. Sperm cryopreservation (usage rate 21–27%) offers a chance of conception, with a pregnancy rate between 33% and 61%. Ovarian tissue cryopreservation is the most promising fertility preservation technique offered in referral centers with experience to patients younger than 35 years, with no contraindications and urgent treatments needed to start. Instead, mature oocyte cryopreservation often leads to delays in treatment commencement (an average of 22 days from first consultation to start of CT). As few data are available on the proportion of patients who opt for cryopreservation and their actual utilization rate, prospective registries should be created to better describe the potential of this technique [8].

The protective role of GnRH analogues in fertility preservation in female lymphoma patients remains controversial. Its use should not be considered an alternative option for fertility preservation but an attempt to reduce the risk of CT-induced ovarian failure. Sperm count, FSH level and the inhibin-B/FSH ratio are appropriate tools to investigate fertility in male patients; serum anti-Müllerian hormone level was also found to be a reliable biomarker of CT-induced ovarian toxicity. Research focused on the optimal time interval from the end of cancer treatment to conception has not been successful in clarifying this. For females, it is currently suggested pregnancy be avoided in the first two to three years from the end of treatment, the period of the greatest risk of lymphoma recurrence. For males, a semen analysis is recommended to exclude serious alterations in spermatogenesis at least one year after the end of treatment before attempting spontaneous conception. Further research into the field of fertility preservation in lymphoma patients is still needed to better define preservation of fertility, the optimal timing of gonadal function assessment and ovarian function biomarkers.

A systematic review focusing on the impact of healthy lifestyles analyzed how to act through tertiary prevention (by means of the adoption of healthy lifestyles) to prevent late CT and RT sequelae for cHL and DLBCL survivors [9]. The following aspects were considered: the impact of physical activity/exercise according to guidelines (at least 150 min/week of moderate physical activity or 75 min/week of intense physical activity) [10], the adoption of the Mediterranean diet or nutritional intervention and the control of body weight and body mass index. Physical activity has been documented to reduce the risk of coronary heart disease (CHD), which is often the first cardiovascular event in a large cohort of young cHL survivors (RR 0.52, with a median interval from lymphoma of 19 years). The same study confirmed obesity in this high-risk population as a predisposing factor for the onset of CHD [11]. Physical exercise can also reduce chronic fatigue in young cHL survivors [12]. Regarding the use of controlled or Mediterranean nutrition, no studies eligible for analysis in the population in question were highlighted.

The results obtained from the data analysis support a change in clinical practice in the management of young cHL survivors, who should be encouraged to engage in physical exercise for at least 150 min/week to improve their QoL and reduce late cardiac toxicities. It is therefore possible to strengthen the recommendation of close collaboration with sports medicine specialists to obtain a structured and effective educational and rehabilitation program [13]. Prospective trials should also include physical activity in outpatient programs of lymphoma survivors and document the impact of structured follow-up on overall outcome. 

Clinical research perspectives on healthy lifestyles in lymphoma survivors are still immature and represent a field in which action can be taken to improve QoL and prevent comorbidities, as well as to allow survivors to benefit from relevant social and economic impacts. The future aim of FIL researchers is to prospectively evaluate an SCP model designed based on the calculated risk of late toxicities and enhanced with indications of healthy lifestyles.

In conclusion, the significant efforts of FIL researchers have highlighted survivorship topics that should be kept in mind during follow-up after lymphoma therapy for cHL and DLBCL survivors. The lack of studies implementing evidence-based guidelines on follow-up procedures to promptly diagnose and manage those late complications should suggest the design of future trials aiming to produce validated statements to improve the quality of life and health of long-term lymphoma survivors.
